# Comparative effectiveness of warfarin, dabigatran, rivaroxaban and apixaban in non-valvular atrial fibrillation: A nationwide pharmacoepidemiological study

**DOI:** 10.1371/journal.pone.0221500

**Published:** 2019-08-26

**Authors:** Lars J. Kjerpeseth, Randi Selmer, Inger Ariansen, Øystein Karlstad, Hanne Ellekjær, Eva Skovlund

**Affiliations:** 1 Department of Public Health and Nursing, Faculty of Medicine and Health Sciences, Norwegian University of Science and Technology (NTNU), Trondheim, Norway; 2 Chronic Diseases and Ageing, Division of Mental and Physical Health, Norwegian Institute of Public Health, Oslo, Norway; 3 Department of Neuromedicine and Movement Science, Faculty of Medicine and Health Sciences, Norwegian University of Science and Technology (NTNU), Trondheim, Norway; 4 Stroke Unit, Department of Internal Medicine, St. Olav’s Hospital, Trondheim, Norway; Institut d'Investigacions Biomediques de Barcelona, SPAIN

## Abstract

**Objective:**

To compare effectiveness and safety of warfarin and the direct oral anticoagulants (DOAC) dabigatran, rivaroxaban and apixaban in non-valvular atrial fibrillation in routine care.

**Methods:**

From nationwide registries, we identified treatment-naïve patients initiating warfarin, dabigatran, rivaroxaban or apixaban for non-valvular atrial fibrillation from July 2013 to December 2015 in Norway. We assessed prescription duration using reverse waiting time distribution. Adjusting for confounding in a Cox proportional hazards model, we estimated one-year risks for ischemic stroke, transient ischemic attack (TIA) or systemic embolism, major or clinically relevant non-major bleeding; intracranial; gastrointestinal; and other bleeding. We censored at switch of treatment or 365 days of follow-up.

**Results:**

We included 30,820 treatment-naïve patients. Compared to warfarin, the adjusted hazard ratios (HR) for ischemic stroke, TIA or systemic embolism were 0.96 (95% CI 0.71–1.28) for dabigatran, 1.12 (95% CI 0.87–1.45) for rivaroxaban and 0.97 (95% CI 0.75–1.26) for apixaban. Corresponding hazard ratios for major or clinically relevant non-major bleeding were 0.73 (95% CI 0.62–0.86) for dabigatran, 0.97 (95% CI 0.84–1.12) for rivaroxaban and 0.71 (95% CI 0.62–0.82) for apixaban. Statistically significant differences of other safety outcomes compared to warfarin were fewer intracranial bleedings with dabigatran (HR 0.28, 95% CI 0.14–0.56), rivaroxaban (HR 0.40, 95% CI 0.23–0.69) and apixaban (HR 0.56, 95% CI 0.34–0.92); fewer gastrointestinal bleedings with apixaban (HR 0.70, 95% CI 0.52–0.93); and fewer other bleedings with dabigatran (HR 0.67, 95% CI 0.55–0.81) and apixaban (HR 0.70, 95% CI 0.59–0.83).

**Conclusion:**

After 1 year follow-up in treatment-naïve patients initiating oral anticoagulation for non-valvular atrial fibrillation, all DOACs were similarly effective as warfarin in prevention of ischemic stroke, TIA or systemic embolism. Safety from bleedings was similar or better, including fewer intracranial bleedings with all direct oral anticoagulants, fewer gastrointestinal bleedings with apixaban and fewer other bleedings with dabigatran and apixaban.

## Introduction

European guidelines recommend prophylactic oral anticoagulation in patients with non-valvular atrial fibrillation who have a moderate to high risk of stroke [1]. Warfarin has been the mainstay for oral anticoagulation, but requires frequent monitoring and dose adjustments due to a narrow therapeutic window and many interactions with food and drugs [2]. In the last decade, easier-to-use direct-acting oral anticoagulants (DOACs) such as dabigatran, rivaroxaban and apixaban have proven as effective and safe as warfarin for stroke prevention in large randomized controlled trials [3–5]. The DOACs have been quickly incorporated in European guidelines on oral anticoagulation in atrial fibrillation [1, 6]. Among users of oral anticoagulation for atrial fibrillation in Norway, we have seen a shift in market shares from complete warfarin coverage in 2010 to a market share of more than 80% DOACs in new users and 50% in prevalent users in 2015 [7, 8]. Other countries have also seen a rapid uptake in use of DOACs for atrial fibrillation [9–16].

These changes in routine clinical care may have huge implications on the public health burden. Atrial fibrillation is common, especially among the elderly, and its prevalence and associated complications are expected to surge in the next decades owing to an ageing population and increase in predisposing risk factors such as obesity, diabetes and unhealthy lifestyle [17]. The introduction of DOACs has increased the drug repertoire, but also complicated decision-making for prescribers and patients considering oral anticoagulation [18]. While each DOAC has been tested against warfarin, head-to-head trials of DOACs are lacking and indirect comparisons of the trials are difficult because of differences in study populations. Also, the standardized approach to treatment and highly selected patients in clinical trials may limit their generalizability. Patients with severe renal impairment were excluded from the DOAC trials and especially dabigatran requires a high renal clearance. Other concerns with regard to applicability of the trial results in routine care are elderly patients with multiple comorbidities, polypharmacy, and compliance issues [1]. Taken together, these issues warrant observational comparative effectiveness studies in everyday clinical practice [19, 20].

Although a number of such studies have been performed, few have compared both dabigatran, rivaroxaban and apixaban separately to warfarin in the same study population [21, 22]. In addition, many of these studies are based on selected populations [21, 22]. In contrast, the cradle-to-grave health registers of Norway allow inclusion of a nationwide patient population from primary and secondary care, and with little loss to follow-up [23]. Furthermore, Norway’s tax-supported universal healthcare system funds nearly all patient expenses and insures equal access to warfarin and the more expensive DOACs [24]. And while Halvorsen et al. investigated the safety of oral anticoagulants in atrial fibrillation using Norwegian registers, they did not address effectiveness outcomes [25]. Thus, we have conducted a study to compare both effectiveness and safety of warfarin, dabigatran, rivaroxaban and apixaban in an unselected and nationwide population in Norway.

## Materials and methods

### Study design

We have performed a register-based cohort study from 15 July 2013 to 31 December 2015. Dabigatran, rivaroxaban and apixaban received Norwegian marketing authorization for non-valvular atrial fibrillation in August 2011, December 2011 and November 2012, respectively. 15 July 2013 was chosen as start date of this study because this was when apixaban, as the last study drug, received general reimbursement for treatment of atrial fibrillation, and reimbursement codes were used to identify patients treated for atrial fibrillation. This also allowed for a run-in period for the DOACs in general and attenuated potential bias from including early adopters of DOACs [26]. We adopted an active comparator, new-user design to reduce prevalent user bias and confounding by indication [27, 28].

### Data sources

Using the personal identification number unique to all Norwegian residents, we linked data from four nationwide databases: the Norwegian Prescription Database, the Norwegian Patient Registry, the Norwegian Cause of Death Registry and the National Registry. The Norwegian Prescription Database contains the dispensing date, Anatomical Therapeutic Chemical (ATC) classification code, package size, tablet strength, and reimbursement code of every prescription claimed since 2004. The indication for use is indicated by the reimbursement codes that since 2008 have been coded according to the International Classification of Diseases, 10th revision (ICD-10) and the International Classification of Primary Care, 2nd Edition (ICPC-2). The Norwegian Patient Registry contains discharge dates and ICD diagnoses of in- and outpatient visits to hospitals and private specialist practices on an individual level since 2008. Up to two primary diagnoses and (potentially) an unlimited number of secondary diagnoses are recorded at each in- and outpatient visit. The Norwegian Cause of Death Registry has records of all deaths since 1951, and uses an international algorithm to ensure uniform assessment of the underlying cause of death and code it according to the ICD coding system. The National Registry is a civil registration system of all residents that includes information on sex, date of birth, and date of emigration, death and other changes in resident status.

### Study population

**Fig 1:**
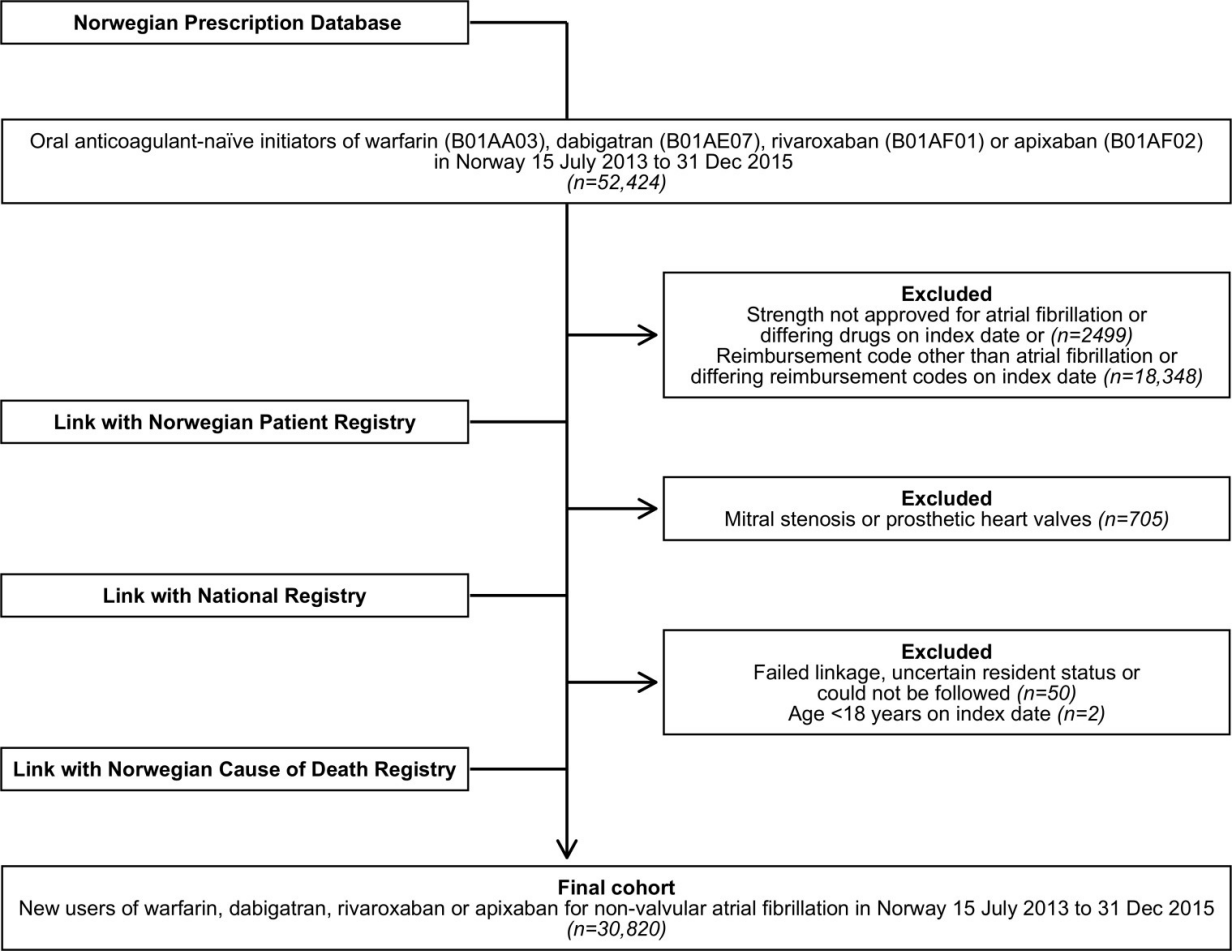
Study population selection. Flowchart showing selection of the final study population of new users of warfarin, dabigatran, rivaroxaban or apixaban for non-valvular atrial fibrillation from 15 July 2013 to 31 December 2015 in Norway by linkage of nationwide registries.

We identified all individuals who had been oral anticoagulant-naïve since 2004 and then received at least one dispensing of a study drug between 15 July 2013 and 31 December 2015. We further restricted the population to adults ≥18 years on the date of the first dispensing who received a reimbursement code for atrial fibrillation/-flutter (ICD-10 code I48 or ICPC-2 code K78) on the first dispensing and had no previous history of mitral stenosis or prosthetic heart valves (‘valvular’ atrial fibrillation) [6]. We excluded individuals who on the date of first dispensing received more than one type of oral anticoagulant or reimbursement code, or a tablet strength not approved for treatment of atrial fibrillation. We also excluded a few patients who failed linkage, had an uncertain resident status or could not be followed, e.g. citizens living abroad (Fig 1 and S1 Table).

### Exposure to oral anticoagulants

We considered a patient’s use of oral anticoagulation to start on the date he or she first received a dispensing for a study drug (index date), and continue as long as the patient received a new dispensing within the duration of the drug supply of the preceding dispensing. We estimated the duration of each dispensing using reverse parametric waiting time distribution [29, 30]. According to a validation study using warfarin as a case, which has a large interindividual variation in daily dose, this approach gives less bias and higher precision than traditional methods assuming a fixed daily dose [31]. The method considers the time from the last dispensing of each patient before an index time point, or reverse waiting time. From the distribution of these reverse waiting times, one can establish maximum likelihood estimates of specified interarrival percentiles, or the time it takes for a percentile of continued users to refill a prescription. If a patient has not redeemed a new prescription within the specified percentile, the treatment is considered to have stopped. Furthermore, the method can take into account covariates predictive of the time from one dispensing to the next, such as age, sex and amount filled. This can reduce misclassification on the individual level [31]. In practice, we pooled all dispensings of a study drug to new users within the last year before 1 November 2015, and used their last filling in this time interval in a reverse parametric waiting time distribution model with 90th percentile interarrival distribution and adjustment for age, sex, and number of tablets dispensed. We repeated the analysis for each study drug separately and used the results to predict dispensing durations for subjects under study. We used 1 November 2015 as the index time point to avoid bias from seasonal stockpiling of study drugs towards the end of the calendar year, which is common in Norway due to the reimbursement rules. We used a log-normal parametric model as this provides a better fit than other models [30]. The analyses can be automated using the publically available w*tdttt* package for Stata.

### Outcomes

We included both primary and secondary diagnoses of the Norwegian Patient Registry in the assessment of outcomes. From the Cause of Death Registry, we included deaths with the outcomes as the underlying cause. Our primary effectiveness outcome was the composite of hospitalizations with a diagnosis of ischemic stroke, transient ischemic attack (TIA) or systemic embolism (arterial thromboembolism outside the brain or lungs), or death from ischemic stroke or systemic embolism. Secondary effectiveness outcomes were hospitalization or death from ischemic stroke or systemic embolism; hospitalization or death from ischemic stroke; and hospitalization with TIA. The primary safety outcome, major or clinically relevant non-major bleeding, was a composite of the secondary safety outcomes, which were intracranial bleeding, gastrointestinal bleeding and other bleeding. We defined intracranial bleeding as hospitalization or death from this diagnosis. Gastrointestinal and other bleeding were similarly defined, with the addition of outpatient diagnoses however, since treatment of such bleedings does not necessarily require hospitalization. Since it can be difficult to discern the cause of intracranial bleedings in register data, we included diagnosis codes of both traumatic and non-traumatic bleedings [32]. Similarly, the ischemic stroke outcome included diagnosis codes of both ischemic and unspecified stroke since the latter will be ischemic in most cases [33]. See S2 Table for further details on diagnosis codes.

### Covariates

We used the patients’ sex, age, county of residence (19 in total), comorbidities, concomitant drug use, the number of hospitalizations (0, 1, ≥2) and the number of outpatient visits (0, 1, ≥2) in the 6 months before index date to adjust for potential confounding. We defined comorbidities from in- and outpatient primary and secondary diagnoses within 5 years before or on index date, and from reimbursement codes for drugs within the same time frame. We considered drugs dispensed within the last 6 months as concomitant drug use. A detailed description of all covariates is found in S3 Table. We calculated CHA_2_DS_2_-VASc score (congestive heart failure, hypertension, age ≥75, diabetes, stroke/TIA/systemic embolism, vascular disease, age 65–74, and sex [female]) and modified HAS-BLED score (hypertension, abnormal liver/kidney function, stroke, bleeding history, age >65, alcohol abuse or concomitant use of non-steroidal anti-inflammatory drugs or platelet inhibitors) for each patient at index date (see S4 Table for calculation of the scores).

### Statistical analyses

Patients were followed from the index date until the first event of the outcome under study, or censoring. We censored patients at switch to another oral anticoagulant; switch to a tablet strength not approved for atrial fibrillation; end of drug supply; death (if not caused by the outcome under study), emigration; 365 days of follow-up; or study end. To avoid bias from differing follow-up times due to differences in the uptake of dabigatran, rivaroxaban and apixaban, we censored follow-up for all study drugs at 365 days. In addition, baseline adjustment of time-varying covariates such as age and co-medications get less valid with longer follow-up. We conducted all analysis in Cox proportional hazards models to estimate hazard ratios (HR) with 95% confidence intervals, after checking the proportional hazards assumption. We report effect estimates for three models in the main analyses and the sensitivity analyses: crude, adjusted for risk factors in the CHA_2_DS_2_-VASc and modified HAS-BLED scores (partially adjusted model), and adjusted for all predefined covariates (fully adjusted model). The results are reported together with median follow-up time, number of events and one-year crude incidence rate for each study drug. In a supplementary analysis, we made plots of the cumulative incidence of each outcome during one year, adjusted for competing risk from deaths not caused by the event (S1 and S3 Figs).

We repeated the primary effectiveness and safety outcome analyses for several subgroups: patients with and without specialist-diagnosed atrial fibrillation; history or no history of vascular disease (i.e. acute myocardioal infarction, atherosclerosis or peripheral vascular disease), stroke/TIA/systemic embolism, serious bleeding, or chronic kidney disease; concomitant or no concomitant use of acetylsalicylic acid or platelet inhibitor; age ≥75 and <75 years; male and female sex; CHA_2_DS_2_-VASc ≥2 and <2; and modified HAS-BLED ≥3 and <3; high-intensity DOAC users; and low-intensity DOAC users. In the high-intensity and low-intensity DOAC subgroup analyses, we respectively compared rivaroxaban 20 mg and apixaban 5 mg to dabigatran 150 mg and rivaroxaban 15 mg and apixaban 2.5 mg to dabigatran 110 mg, and we censored patients who switched to another tablet strength. We only report estimates from the fully adjusted model for the subgroup analyses (S2 and S4 Figs).

We performed several sensitivity analyses on the primary outcomes to test the validity of our results (S5 and S6 Tables). First, we repeated the analyses including only events registered as a primary diagnosis at hospitalization (thereby excluding events registered as secondary diagnoses or underlying cause of death). Second, we repeated the analyses using 50th, 60th, 70th, 80th and 99th interarrival density percentiles in the reverse parametric waiting time distribution model to estimate the duration of dispensings (90th percentile in the main analyses). Third, we repeated the analyses using a fixed dose model where we estimated the duration of a dispensing from the number of tablets dispensed. We assumed a dosing regimen of one tablet twice daily for dabigatran and apixaban and one tablet once daily for rivaroxaban. For warfarin, we calculated a mean daily tablet dose for the study population using the accumulated time from a dispensing of warfarin to the next dispensing as the denominator and the total amount of tablets of warfarin in these dispensings as the nominator. We allowed a 30-day gap in the oral anticoagulant supply (grace period) in this model.

To test how well we were able to control for confounding, we performed a sensitivity analysis using a presumed “neutral” outcome, hospitalization or death from pneumonia, as a negative control [34] (S7 Table). Pneumonia have many overlapping risk factors with stroke and bleeding, such as old age, underlying medical conditions and lifestyle factors [35], which we expect to contribute to confounding since they are known to influence the choice of oral anticoagulant in atrial fibrillation [8, 36, 37]. Channeling of patients towards specific study drugs based on these risk factors would lead to differences in the risk of pneumonia associated with each drug. A similar risk of pneumonia between drug groups after adjustment for covariates would thus provide indirect evidence of no residual confounding.

We used Stata/SE version 15.0 to analyze the data and considered two-sided p-values <0.05 as statistically significant. The South East Regional Committee for Medical and Health Research Ethics approved the study and granted a waiver of the requirement for obtaining patient consent (2010/131). The Norwegian Data Protection Authority gave a license to link registry data (13/00577-4/CGN). Reporting conform to the Strengthening the Reporting of Observational Studies in Epidemiology (STROBE) statement (S1 Checklist).

## Results

We identified 30,820 new users of warfarin, dabigatran, rivaroxaban or apixaban for non-valvular atrial fibrillation from 15 July 2013 to 31 December 2015 (Table 1). Sixty-nine percent had previously been diagnosed with atrial fibrillation during hospitalization or an outpatient visit, and 12% had valvular disease such as mitral/aortic/tricuspid insufficiency or aortic/tricuspid stenosis (patients with mitral stenosis or heart valve prosthesis had been excluded from the study cohort). Their mean age was 73.3 years and 47% were elderly (≥75 years). Women constituted 44% of the cohort. Eighty-four percent had a CHA_2_DS_2_-VASc score of ≥2, and 47% a modified HAS-BLED score of ≥3. Fifteen percent had 15 or more prescription drugs dispensed in the last 6 months before starting oral anticoagulation. Initiators of warfarin or apixaban were generally older and had more comorbidities and higher scores on CHA_2_DS_2_-VASc and modified HAS-BLED than users of dabigatran or rivaroxaban.

**Table 1:** Baseline characteristics of non-valvular atrial fibrillation patients initiating warfarin, dabigatran, rivaroxaban or apixaban for non-valvular atrial fibrillation from 15 July 2013 to 31 December 2015 in Norway. Values are numbers (percentages) unless stated otherwise.

Characteristics	Warfarin	Dabigatran	Rivaroxaban	Apixaban	Total
Number of patients	6435 (100)	5984 (100)	7851 (100)	10550 (100)	30820 (100)
Year at index date					
2013	1747 (27)	1753 (29)	1414 (18)	350 (3)	5264 (17)
2014	2961 (46)	3076 (51)	3048 (39)	3401 (32)	12486 (41)
2015	1727 (27)	1155 (19)	3389 (43)	6799 (64)	13070 (42)
Age, years					
Mean (SD)	73.6 (11.9)	70.6 (11.3)	73.7 (10.9)	74.2 (11.0)	73.3 (11.3)
18–64	1241 (19)	1517 (25)	1398 (18)	1761 (17)	5917 (19)
65–74	1876 (29)	2249 (38)	2659 (34)	3488 (33)	10272 (33)
≥75	3318 (52)	2218 (37)	3794 (48)	5301 (50)	14631 (47)
Female	2629 (41)	2370 (40)	3553 (45)	4876 (46)	13428 (44)
Oral anticoagulant initiated by physician in secondary or tertiary care	3323 (52)	3312 (55)	4174 (53)	7290 (69)	18099 (59)
Low-intensity DOAC	-	2038 (34)	2085 (27)	2877 (27)	7000 (23)
***Medical history (last 5 years)***					
Atrial fibrillation previously diagnosed in secondary or tertiary care	4478 (70)	3821 (64)	5055 (64)	7788 (74)	21142 (69)
Pacemaker / defibrillator	289 (4)	228 (4)	362 (5)	493 (5)	1372 (4)
Valvular disease (÷ mitral stenosis or heart valve prosthesis)	954 (15)	545 (9)	895 (11)	1383 (13)	3777 (12)
Congestive heart failure	2300 (36)	1295 (22)	1930 (25)	3138 (30)	8663 (28)
Hypertension	4591 (71)	3867 (65)	5529 (70)	7581 (72)	21568 (70)
Diabetes mellitus	1144 (18)	748 (13)	1107 (14)	1613 (15)	4612 (15)
Ischemic stroke, TIA or systemic embolism	895 (14)	781 (13)	1281 (16)	1885 (18)	4842 (16)
History of acute myocardial infarction	1477 (23)	714 (12)	1048 (13)	1641 (16)	4880 (16)
Atherosclerosis or peripheral artery disease	472 (7)	274 (5)	517 (7)	736 (7)	1999 (6)
Chronic kidney disease	1031 (16)	279 (5)	546 (7)	1094 (10)	2950 (10)
Liver disease	163 (3)	109 (2)	156 (2)	189 (2)	617 (2)
History of intracranial bleeding	66 (1)	35 (<1)	61 (<1)	99 (<1)	261 (<1)
History of gastrointestinal bleeding	278 (4)	162 (3)	217 (3)	357 (3)	1014 (3)
History of other major bleeding	626 (10)	432 (7)	689 (9)	971 (9)	2718 (9)
Alcohol misuse	89 (1)	96 (2)	143 (2)	162 (2)	490 (2)
Chronic lung disease	1565 (24)	1277 (21)	1792 (23)	2676 (25)	7310 (24)
Cancer	835 (13)	572 (10)	924 (12)	1270 (12)	3601 (12)
Coagulation/platelet defect	163 (3)	120 (2)	163 (2)	183 (2)	629 (2)
Venous thromboembolism	191 (3)	95 (2)	234 (3)	232 (2)	752 (2)
Disease in precerebral or cerebral artery	190 (3)	145 (2)	197 (3)	313 (3)	845 (3)
Esophagitis, gastritis, duodenitis, acid reflux or peptic ulcer	1744 (27)	1141 (19)	1769 (23)	2586 (25)	7240 (23)
Inflammatory bowel disease	96 (1)	72 (1)	110 (1)	137 (1)	415 (1)
Osteoporosis	531 (8)	381 (6)	627 (8)	1018 (10)	2557 (8)
Anemia	975 (15)	536 (9)	883 (11)	1298 (12)	3692 (12)
Undernourished or vitamin deficiency	326 (5)	199 (3)	360 (5)	541 (5)	1426 (5)
Dementia	127 (2)	86 (1)	188 (2)	255 (2)	656 (2)
Delirium	67 (1)	27 (<1)	94 (1)	144 (1)	332 (1)
Fall	245 (4)	212 (4)	297 (4)	471 (4)	1225 (4)
Number of hospital admissions in last 6 months					
0	2431 (38)	3090 (52)	4063 (52)	4051 (38)	13635 (44)
1	2586 (40)	2258 (38)	2833 (36)	4849 (46)	12526 (41)
≥2	1418 (22)	636 (11)	955 (12)	1650 (16)	4659 (15)
Number of outpatient visit in last 6 months					
0	2103 (33)	2153 (36)	2534 (32)	3296 (31)	10086 (33)
1	1464 (23)	1480 (25)	1807 (23)	2571 (24)	7322 (24)
≥2	2868 (45)	2351 (39)	3510 (45)	4683 (44)	13412 (44)
***Prescription drug use (last 6 months)***					
Number of drugs dispensed					
0–4	889 (14)	1248 (21)	1259 (16)	1521 (14)	4917 (16)
5–9	2427 (38)	2733 (46)	3394 (43)	4400 (42)	12954 (42)
10–14	1874 (29)	1408 (24)	2136 (27)	2968 (28)	8386 (27)
≥15	1245 (19)	595 (10)	1062 (14)	1661 (16)	4563 (15)
Antacids, H2-receptor antagonist or PPI	1952 (30)	1226 (20)	1961 (25)	2860 (27)	7999 (26)
Nonsteroidal anti-inflammatory drug	1405 (22)	1430 (24)	1828 (23)	2453 (23)	7116 (23)
Glucocorticoids	1047 (16)	650 (11)	1018 (13)	1407 (13)	4122 (13)
Acetylsalicylic acid	3617 (56)	2641 (44)	3957 (50)	5311 (50)	15526 (50)
Platelet aggregation inhibitor	1011 (16)	487 (8)	770 (10)	1208 (11)	3476 (11)
ACE inhibitor or angiotensin II antagonist	3518 (55)	2891 (48)	3979 (51)	5568 (53)	15956 (52)
Antiarrhythmic	654 (10)	310 (5)	426 (5)	819 (8)	2209 (7)
Beta blocking agent	4852 (75)	4237 (71)	5322 (68)	7671 (73)	22082 (72)
Calcium channel blocker	1906 (30)	1433 (24)	2091 (27)	2921 (28)	8351 (27)
Digitoxin / digoxin	385 (6)	293 (5)	329 (4)	556 (5)	1563 (5)
Diuretics	2297 (36)	1233 (21)	1934 (25)	2892 (27)	8356 (27)
Lipid modifying agent	3267 (51)	2468 (41)	3544 (45)	5096 (48)	14375 (47)
Nitrate	916 (14)	459 (8)	746 (10)	1219 (12)	3340 (11)
Antidepressant	700 (11)	577 (10)	893 (11)	1244 (12)	3414 (11)
Antipsychotic	218 (3)	164 (3)	275 (4)	349 (3)	1006 (3)
Anxiolytic, hypnotic or sedative	2140 (33)	1746 (29)	2537 (32)	3464 (33)	9887 (32)
Antiepileptic	297 (5)	210 (4)	342 (4)	426 (4)	1275 (4)
***Risk scores***					
CHA_2_DS_2_-VASc					
≥2	5542 (86)	4730 (79)	6812 (87)	9326 (88)	26410 (86)
Mean (SD)	3.5 (1.8)	2.9 (1.7)	3.4 (1.7)	3.5 (1.7)	3.4 (1.7)
Modified HAS-BLED					
≥3	3554 (55)	2513 (42)	3968 (51)	5689 (54)	15724 (51)
Mean (SD)	2.6 (1.2)	2.2 (1.2)	2.5 (1.1)	2.5 (1.1)	2.5 (1.2)

TIA: transient ischemic attack. PPI: proton pump inhibitor. CHA_2_DS_2_-VASc: congestive heart failure, hypertension, age ≥75 [doubled], previous stroke/TIA/systemic embolism [doubled], vascular disease, age 65–74, female sex. Modified HAS-BLED: hypertension, abnormal liver/kidney function, previous stroke/TIA/systemic embolism, bleeding history, age >65, alcohol abuse or concomitant use of non-steroidal anti-inflammatory drugs or platelet inhibitor.

Between study entry and study end on 31 December 2015, 1965 patients (6.4%) died; 649 (2.1%) had an ischemic stroke; 271 (0.9%) had a TIA; 42 (0.1%) had a systemic embolism; 246 (0.8%) had an intracranial bleeding; 812 (2.6%) had a gastrointestinal bleeding; and 2091 (6.8%) had a bleeding in another location. For all outcomes in the main analyses, median follow-up was longest for warfarin users and shortest for rivaroxaban users and apixaban users (Table 2).

**Table 2:** Median days of follow-up, number of events, crude incidence rate per 100 person years and hazard ratio of effectiveness and safety outcomes in new users of dabigatran, rivaroxaban or apixaban compared to warfarin for non-valvular atrial fibrillation.

Outcome	Oral anticoagulant	Median days of follow-up (min-max)	No. of events	Incidence rate (per 100 person years)	Hazard ratio (95% confidence interval)^§^		
					Crude	Partially adjusted	Fully adjusted
**Ischemic stroke, TIA or systemic embolism**	Warfarin	248 (1–365)	124	2.99	1	1	1
	Dabigatran	211 (1–365)	88	2.48	0.80	0.92 (0.69–1.21)	0.96 (0.71–1.28)
	Rivaroxaban	164 (1–365)	140	3.48	1.08	1.09 (0.85–1.40)	1.12 (0.87–1.45)
	Apixaban	150 (1–365)	180	3.56	1.06	1.00 (0.79–1.28)	0.97 (0.75–1.26)
**Ischemic stroke or systemic embolism**	Warfarin	250 (1–365)	91	2.19	1	1	1
	Dabigatran	212 (1–365)	62	1.74	0.77	0.86 (0.62–1.19)	0.90 (0.64–1.27)
	Rivaroxaban	164 (1–365)	111	2.75	1.16	1.14 (0.86–1.51)	1.23 (0.91–1.65)
	Apixaban	150 (1–365)	144	2.84	1.14	1.02 (0.77–1.35)	1.00 (0.74–1.35)
**Ischemic stroke**	Warfarin	250 (1–365)	84	2.02	1	1	1
	Dabigatran	212 (1–365)	60	1.68	0.80	0.89 (0.63–1.25)	0.93 (0.65–1.32)
	Rivaroxaban	164 (1–365)	104	2.58	1.17	1.14 (0.85–1.53)	1.22 (0.90–1.66)
	Apixaban	150 (1–365)	141	2.78	1.20	1.07 (0.81–1.43)	1.05 (0.77–1.42)
**Transient ischemic attack**	Warfarin	251 (1–365)	35	0.84	1	1	1
	Dabigatran	213 (1–365)	28	0.78	0.92	1.12 (0.67–1.86)	1.11 (0.65–1.90)
	Rivaroxaban	166 (1–365)	33	0.81	0.93	1.00 (0.61–1.62)	0.89 (0.54–1.48)
	Apixaban	152 (1–365)	41	0.80	0.90	0.96 (0.59–1.55)	0.87 (0.52–1.45)
**Major or clinically relevant non-major bleeding**	Warfarin	237 (1–365)	490	12.19	1	1	1
	Dabigatran	205 (1–365)	245	6.99	0.57	0.71 (0.60–0.83)	0.73 (0.62–0.86)
	Rivaroxaban	155 (1–365)	412	10.45	0.84	0.95 (0.83–1.08)	0.97 (0.84–1.12)
	Apixaban	148 (1–365)	446	8.94	0.70	0.73 (0.63–0.83)	0.71 (0.62–0.82)
**Intracranial bleeding**	Warfarin	252 (1–365)	48	1.15	1	1	1
	Dabigatran	214 (1–365)	11	0.31	0.27	0.31 (0.16–0.60)	0.28 (0.14–0.56)
	Rivaroxaban	167 (1–365)	20	0.49	0.42	0.45 (0.27–0.77)	0.40 (0.23–0.69)
	Apixaban	152 (1–365)	37	0.72	0.61	0.66 (0.41–1.05)	0.56 (0.34–0.92)
**Gastrointestinal bleeding**	Warfarin	248 (1–365)	124	2.98	1	1	1
	Dabigatran	212 (1–365)	84	2.36	0.78	1.04 (0.79–1.39)	1.10 (0.82–1.48)
	Rivaroxaban	164 (1–365)	121	3.00	0.97	1.15 (0.89–1.49)	1.21 (0.92–1.59)
	Apixaban	151 (1–365)	112	2.20	0.70	0.71 (0.55–0.94)	0.70 (0.52–0.93)
**Other bleeding**	Warfarin	240 (1–365)	357	8.80	1	1	1
	Dabigatran	207 (1–365)	164	4.65	0.52	0.65 (0.53–0.78)	0.67 (0.55–0.81)
	Rivaroxaban	157 (1–365)	311	7.84	0.87	0.98 (0.84–1.15)	1.02 (0.87–1.20)
	Apixaban	149 (1–365)	317	6.32	0.69	0.72 (0.61–0.84)	0.70 (0.59–0.83)

CHA_2_DS_2_-VASc: congestive heart failure, hypertension, age ≥75 [doubled], previous stroke/TIA/systemic embolism [doubled], vascular disease, age 65–74, female sex. Modified HAS-BLED: hypertension, abnormal liver/kidney function, previous stroke/TIA/systemic embolism, bleeding history, age >65, alcohol abuse or concomitant use of non-steroidal anti-inflammatory drugs or platelet inhibitor.

§: Hazard ratios for the risk of an outcome for dabigatran, rivaroxaban or apixaban compared to warfarin in Cox proportional hazard models with no adjustment (crude); adjusted for year and risk factors of CHA_2_DS_2_-VASc and modified HAS-BLED (partially adjusted); and adjusted for all predefined covariates (fully adjusted).

**Fig 2:**
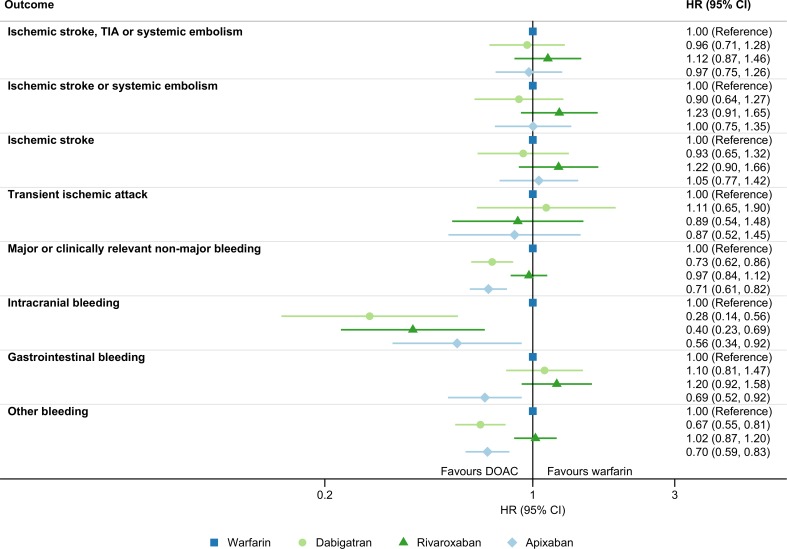
Effectiveness and safety of DOACs vs warfarin in non-valvular atrial fibrillation. Adjusted one-year hazard ratio (HR) and 95% confidence interval (CI) for effectiveness and safety outcomes in new users of direct oral anticoagulants compared to warfarin for non-valvular atrial fibrillation in Norway July 2013 to December 2015.

Cumulative incidence curves for the effectiveness outcomes are presented in S1 Fig. After adjustment for all covariates, the risk of ischemic stroke, TIA or systemic embolism differed little from warfarin for both dabigatran, rivaroxaban and apixaban (Table 2 and Fig 2). Although the estimates were imprecise, we observed similar results in the subgroup analyses, including the high- and low-intensity DOAC subgroups were the risk of the primary effectiveness outcome did not differ for rivaroxaban or apixaban compared to dabigatran (S2 Fig). Likewise, the risks were similar compared to warfarin for the secondary effectiveness outcomes ischemic stroke or systemic embolism; ischemic stroke; and TIA (Table 2 and Fig 2).

Cumulative incidence curves for the safety outcomes are presented in S3 Fig. Compared to warfarin, dabigatran and apixaban were superior with regard to the risk of major or clinically relevant non-major bleeding after adjustment for all covariates, while patients receiving rivaroxaban had a similar risk (Table 2 and Fig 2). We observed the same trend for the direction of the hazard ratios in all subgroups comparing DOACs to warfarin, although it was less clear among patients with a history of stroke/TIA/systemic embolism, major bleeding, or chronic kidney disease (S4 Fig). Compared to dabigatran, rivaroxaban use was associated with a higher risk of major or clinically relevant non-major bleeding in the high-intensity DOAC subgroup, while the risk was similar with rivaroxaban in the low-intensity DOAC subgroup and with apixaban in both DOAC subgroups (S4 Fig).

The crude incident rate for intracranial bleeding was higher with use of warfarin than any DOAC, and the risk for this outcome remained lower for all three DOACs after adjustment for all covariates. The risk of gastrointestinal bleeding was comparable to warfarin for both dabigatran and rivaroxaban. In contrast, the risk was lower in apixaban users than warfarin users. With regard to bleedings in locations other than the intracranial and gastrointestinal cavities, the risk compared to those using warfarin was lower for patients using dabigatran or apixaban, and similar for rivaroxaban users (Table 2 and Fig 2).

The sensitivity analysis on the primary outcomes using the fixed dose model and the reverse parametric waiting time distribution model with different percentiles of interarrival density yielded similar results as the main analyses (S5 and S6 Tables). The risk of ischemic stroke, TIA or systemic embolism with rivaroxaban compared to warfarin was higher with the fixed dose model however, and reached statistical significance. Using only primary diagnosis events in the effectiveness outcome resulted in a lower risk for the primary effectiveness outcome with all three DOACs compared to warfarin, the estimates were uncertain due to fewer total events however. Including only primary diagnosis events in the primary safety outcome did not markedly change the results from the main analysis. In the analysis on the negative control outcome, we observed a lower crude risk of hospitalization or death from pneumonia with all DOACs compared to warfarin. The difference diminished with increasing adjustment for covariates, although not completely (S7 Table).

## Discussion

Comparing the DOACs dabigatran, rivaroxaban or apixaban to warfarin, we did not find any decisive differences in the risk of ischemic stroke, TIA or systemic embolism; ischemic stroke or systemic embolism; ischemic stroke; and TIA. Small differences could not be excluded however. Furthermore, in the current study dabigatran and apixaban were superior to warfarin for the risk of major or clinically relevant non-major bleeding, while the risk was similar with rivaroxaban. We found that the risk of intracranial bleeding was lower with all three DOACs than warfarin however. With regard to gastrointestinal bleeding, we observed a lower risk with apixaban than warfarin and comparable risk with dabigatran or rivaroxaban, while other bleedings were less common with dabigatran or apixaban than warfarin and similar with rivaroxaban.

Similar findings have been observed in both randomized controlled trials and observational studies. In the trials, the risk of ischemic or unspecified stroke did not differ from warfarin with rivaroxaban or apixaban. For dabigatran, the risk was lower with the high-intensity dose and numerically higher with low-intensity dose in the RE-LY trial, which is consistent with no net better efficacy when we compared both regimens to warfarin in the present study. A meta-analysis of 28 observational studies reported similar rates of ischemic stroke and ischemic stroke or systemic embolism with all three DOACs compared to vitamin K antagonists [21]. In accordance with our results, a Swedish study found comparable risks between all three DOACs and warfarin for the combined outcome of TIA and ischemic and unspecified stroke [38]. With regard to TIA as an individual outcome, we observed no differences in risk between individual DOACs and warfarin. A German claims-based study found an increased risk of TIA in atrial fibrillation for DOACs combined compared to vitamin K antagonists [39]. However, in the latter group >99% received phenprocoumon, which differs pharmacokinetically from warfarin [39]. The DOAC trials did not report this outcome [3–5].

The aforementioned meta-analysis reported similar rates of major bleeding with rivaroxaban and fewer with apixaban compared to vitamin K antagonists; more gastrointestinal bleedings with dabigatran and rivaroxaban and fewer with apixaban; and a large reduction in intracranial bleeding with all three DOACs. It also suggested a lower risk of major bleeding with dabigatran than warfarin, although the result was not statistically significant. These results are in line with the current study: Compared to warfarin, we found the risk of major or clinically relevant non-major bleeding to be 27% lower with dabigatran, similar with rivaroxaban, and 30% lower with apixaban; while the risk of gastrointestinal bleeding was numerically higher with dabigatran and rivaroxaban, and significantly lower with apixaban. In randomized controlled trials, the risk of major or minor bleeding was 9% lower with high-intensity dabigatran and 22% lower with low-intensity dabigatran compared to warfarin; the risk of major or clinically relevant non-major bleeding did not differ with rivaroxaban and was 32% lower with apixaban; and there were more gastrointestinal bleedings with rivaroxaban and both regimens of dabigatran and fewer with apixaban [3–5].

A lower risk of intracranial bleeding with DOACs compared to warfarin has been reported consistently in all randomized controlled trials [3–5, 40], a finding which is also replicated in the present study. The reason for the relatively higher risk of intracranial bleeding with warfarin is unclear. A possible explanation could be disturbance of the hemostasis by inhibition of coagulation factor VIIa, which forms complexes with tissue factor, highly expressed in brain vessels, and together is a key initiator of the coagulation cascade [41]. In another nationwide Norwegian registry study, Gulati et al. found increased risks of intracranial bleeding with warfarin, rivaroxaban and apixaban compared to no antithrombotic use, while the risk did not increase with dabigatran [42]. This finding is consistent with a particularly low risk of intracranial bleeding with dabigatran in the current study.

With regard to bleeding in other locations than the intracranial and gastrointestinal cavities, the risk was similar with rivaroxaban compared to warfarin, and significantly lower with dabigatran and apixaban. This is consistent with findings in several other observational studies [25, 43–45] and the ARISTOTLE trial [5]. The RE-LY and ROCKET AF trials did not report this outcome. A meta-analysis of observational studies comparing dabigatran, rivaroxaban and apixaban found that the latter had the best safety profile. Our results also point in this direction since, unlike the other DOACs, the risk for all three subtypes of bleeding was lower with apixaban than warfarin. The current study also gives credence to studies that find DOACs, especially dabigatran and apixaban, to be more cost-effective than warfarin [46]. While DOACs do not seem to be more effective than warfarin with regard to stroke prevention, their uptake was followed by fewer ischemic strokes but no more bleedings in a regional Swedish atrial fibrillation population [47]. This might be mediated through a lowered threshold of oral anticoagulation coupled with a lower bleeding risk with DOACs. The trend was also present before their introduction however [48]. Interestingly, our results on arterial thromboembolism and bleeding are consistent with the major randomized controlled trials comparing DOACs and warfarin despite the reportedly high quality of anticoagulation treatment with warfarin in Norway [49–52]. However, this is in line with post-hoc analyses indicating that the results of the major clinical trials are consistent across a wide range of warfarin treatment qualities, although any advantages of the DOAC lessens with increasing warfarin treatment quality [49–51].

Using the reverse parametric waiting time distribution model to estimate prescription length, we could define continuous use based on how long it takes for a given percentile of users to refill a prescription. As shown in S5 and S6 Tables, changing the percentiles to 50, 60, 70, 80 or 99 did not markedly change the results for the primary effectiveness and safety outcomes. Using a fixed daily dose and allowing up to 30 days gap in treatment yielded similar results except for a lower effectiveness of rivaroxaban compared to warfarin.

A strength of the present study is the use of reimbursement codes to identify atrial fibrillation as an indication for oral anticoagulation regardless of the level of care the patient receives. While these reimbursement codes have not been validated, 69% of the study population had an atrial fibrillation diagnosis in the Norwegian Patient Registry at the initiation of oral anticoagulation and 80% within 3 months after initiation, and this diagnosis has a positive predictive value of 89% [53]. Restricting the analyses to patients with a confirmed atrial fibrillation diagnosis at baseline did not change the conclusion with regard to the primary outcomes (S2 and S3 Figs). Apart from atrial fibrillation, few validation studies of diagnoses in the Norwegian Patient Registry exist. However, the diagnosis of acute stroke (ischemic and hemorrhagic) and intracranial bleeding (traumatic and non-traumatic) have been found to be of adequate quality for epidemiological studies [32, 54]. To improve the validity of these outcomes even further in the current study, we included only events that required hospitalization. Linking with the Norwegian Cause of Death Registry we were also able to include fatal events that were otherwise not detected. Reassuringly, the sensitivity analyses using only primary diagnosis events in the outcomes did not lead to different conclusions than the main analyses.

We believe the inclusion of routine care patients from nationwide and mandatory registers in Norway, where healthcare is universal and affordable, ensure generalizability of our results to other atrial fibrillation populations with a similar constitution. While we did not have information on ethnicity or race, and Norway has become more multiethnic in recent decades, the elderly population constituting most atrial fibrillation patients is likely predominantly white (95% of the cohort were born in Norway). Likewise, we did not have information on other potentially prognostic factors for physicians choosing between different drugs for specific patients, such as body mass index, consumption of alcohol and tobacco, creatinine clearance and socioeconomic status.

The higher crude rate of hospitalization or death from pneumonia suggests that initiators of warfarin were frailer than DOAC initiators, especially those initiating dabigatran and rivaroxaban. Upon adjusting for additional covariates, the adjusted hazard for DOAC users moved incrementally closer to that of warfarin users, but it still remained lower than for warfarin with all three DOACs in the fully adjusted model (S7 Table). Thus, channeling of elderly and less healthy patients towards warfarin and to some degree apixaban may have generated some residual confounding. Still, we do not believe this largely impacted our main results as they are consistent in subgroups, sensitivity analyses and with trials and other observational studies.

## Conclusion

After 1 year of follow-up in new users, all DOACs were similarly effective as warfarin in prevention of ischemic stroke, TIA or systemic embolism, while safety from bleedings was similar or better, including fewer intracranial bleedings with all DOACs, fewer gastrointestinal bleedings with apixaban and fewer other bleedings with dabigatran and apixaban.

## Supporting information

S1 Checklist STROBE checklist of items that should be included in reports of cohort studies. (PDF)

S1 Fig Cumulative incidence of effectiveness outcomes. (PDF)

S2 Fig Risk of primary effectiveness outcome in subgroups. (PDF)

S3 Fig Cumulative incidence of safety outcomes. (PDF)

S4 Fig Risk of primary safety outcome in subgroups. (PDF)

S1 Table Definition of inclusion and exclusion criteria. (PDF)

S2 Table Definition of study outcomes. (PDF)

S3 Table Definition of covariates. (PDF)

S4 Table Calculation of CHA_2_DS_2_-VASc and modified HAS-BLED risk scores from variables in S3 Table. (PDF)

S5 Table Results of sensitivity analyses on primary effectiveness outcome. (PDF)

S6 Table Results of sensitivity analyses on primary safety outcome. (PDF)

S7 Table Results of sensitivity analysis on negative control outcome. (PDF)
