# Supplementary material for: Comparative effectiveness of warfarin, dabigatran, rivaroxaban and apixaban in non-valvular atrial fibrillation: A nationwide pharmacoepidemiological study
Source: PLoS One. 2019 Aug 26;14(8):e0221500. doi: 10.1371/journal.pone.0221500 (PMC6709911; doi:10.1371/journal.pone.0221500)
Supplement: S1 Fig — (PDF) [file pone.0221500.s002.pdf]

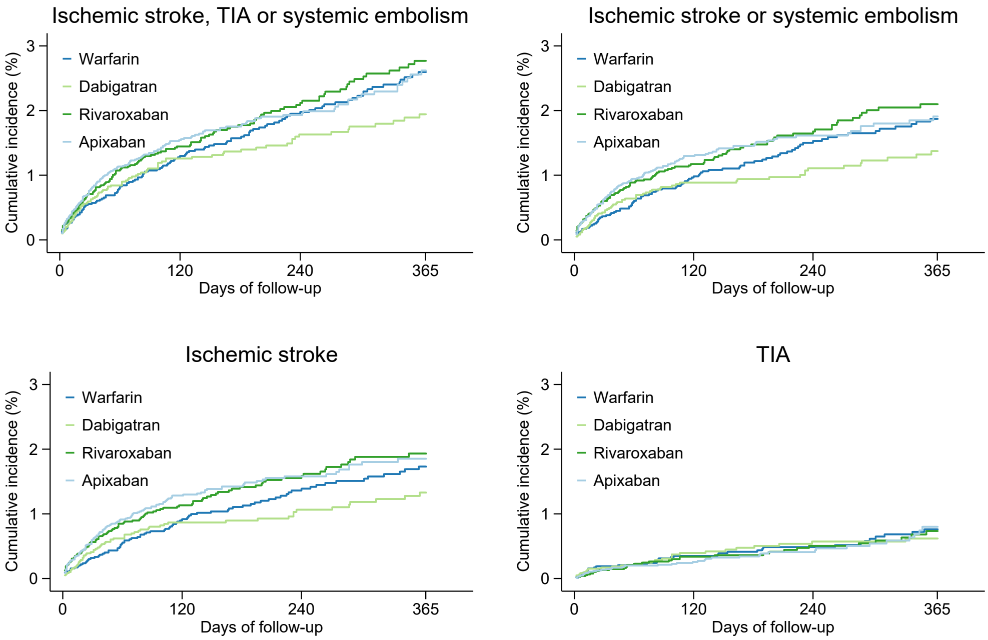

S1 Fig. Cumulative incidence of effectiveness outcomes Cumulative incidence of effectiveness outcomes associated with use of oral anticoagulants for non-valvular atrial fibrillation during one year of follow-up in Norway between 15 July 2013 and 31 December 2015. TIA, transient ischemic attack.
