# Supplementary material for: Comparative effectiveness of warfarin, dabigatran, rivaroxaban and apixaban in non-valvular atrial fibrillation: A nationwide pharmacoepidemiological study
Source: PLoS One. 2019 Aug 26;14(8):e0221500. doi: 10.1371/journal.pone.0221500 (PMC6709911; doi:10.1371/journal.pone.0221500)
Supplement: S2 Fig — (PDF) [file pone.0221500.s003.pdf]

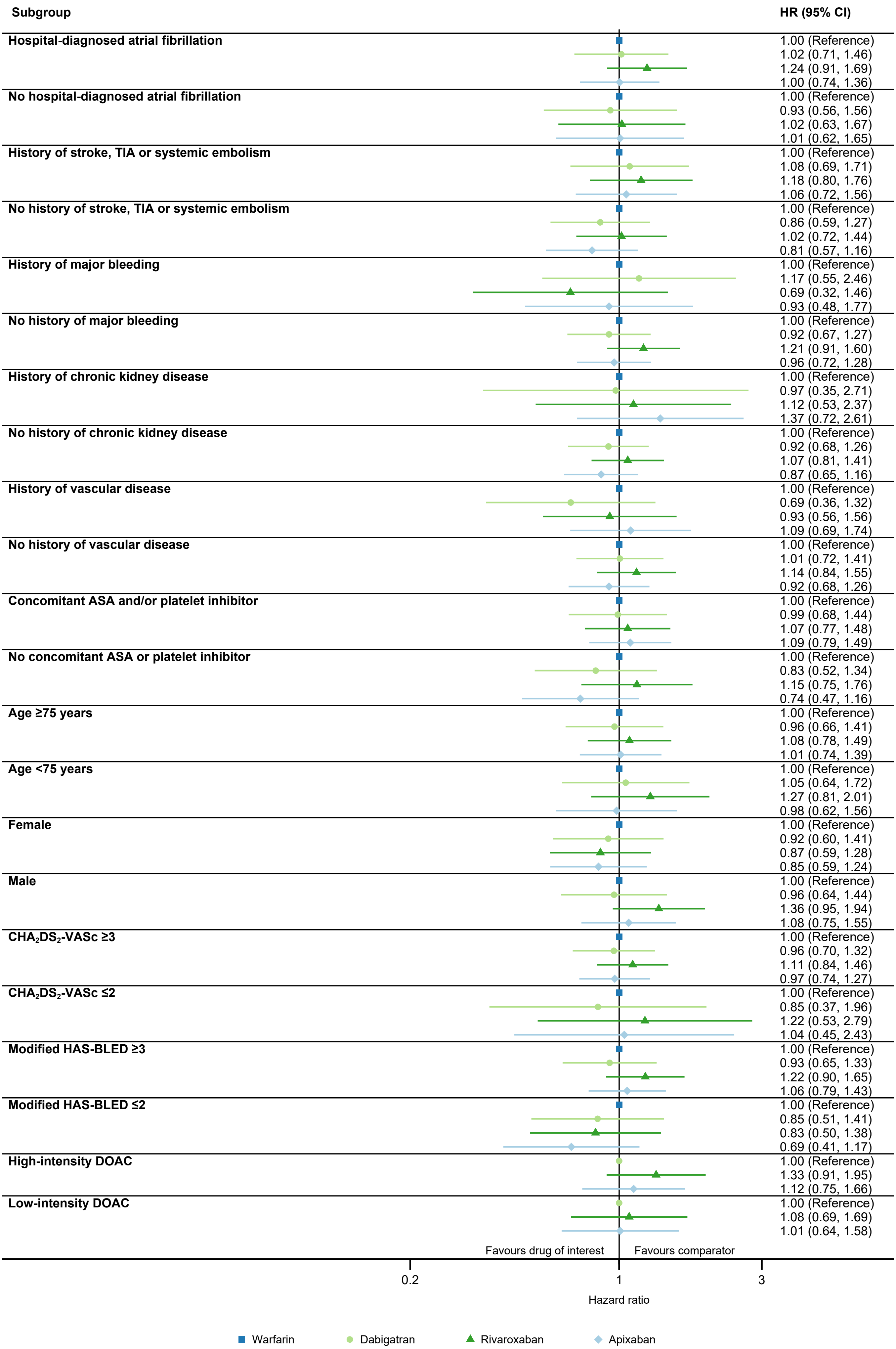

S2 Fig. Risk of primary effectiveness outcome in subgroups Hazard ratios (HR) and 95% confidence intervals (CI) adjusted for sex, age and comorbidities for the risk of ischemic stroke, transient ischemic attack (TIA) or systemic embolism in subgroups of new users of warfarin, dabigatran, rivaroxaban or apixaban for non-valvular atrial fibrillation. The comparator is dabigatran in the high- and low-intensity DOAC subgroups, otherwise warfarin. Norway, 15 July 2013 to 31 December 2015. ASA, acetylsalicylic acid.
