# Supplementary material for: Comparative effectiveness of warfarin, dabigatran, rivaroxaban and apixaban in non-valvular atrial fibrillation: A nationwide pharmacoepidemiological study
Source: PLoS One. 2019 Aug 26;14(8):e0221500. doi: 10.1371/journal.pone.0221500 (PMC6709911; doi:10.1371/journal.pone.0221500)
Supplement: S4 Fig — (PDF) [file pone.0221500.s005.pdf]

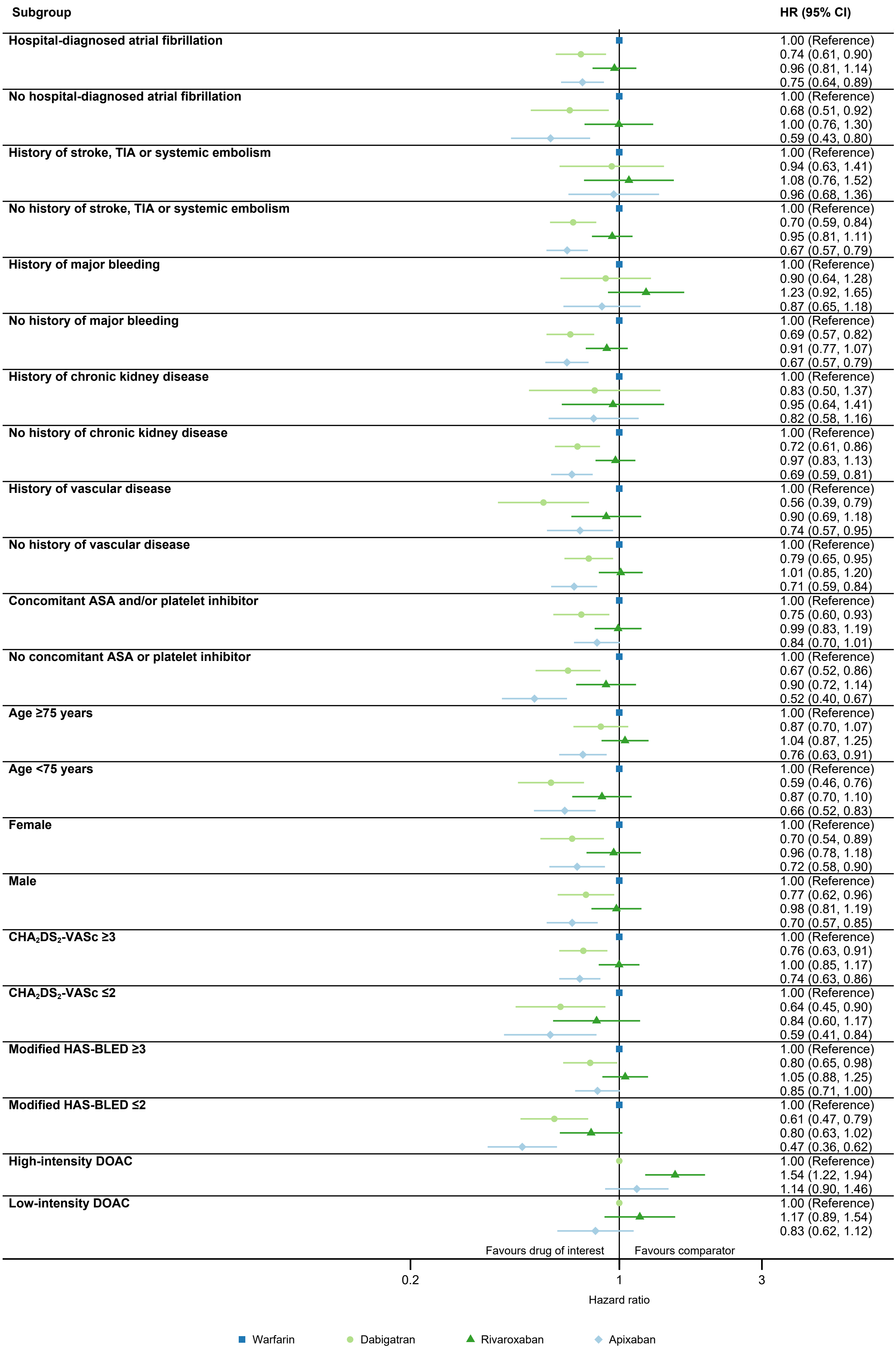

S4 Fig. Risk of primary safety outcome in subgroups Hazard ratios (HR) and 95% confidence intervals (CI) adjusted for sex, age and comorbidities for the risk of major or clinically relevant non-major bleeding in subgroups of new users of warfarin, dabigatran, rivaroxaban or apixaban for non-valvular atrial fibrillation. The comparator is dabigatran in the high- and low-intensity DOAC subgroups, otherwise warfarin. Norway, 15 July 2013 to 31 December 2015. ASA, acetylsalicylic acid.
