# Supplementary material for: Comparative effectiveness of warfarin, dabigatran, rivaroxaban and apixaban in non-valvular atrial fibrillation: A nationwide pharmacoepidemiological study
Source: PLoS One. 2019 Aug 26;14(8):e0221500. doi: 10.1371/journal.pone.0221500 (PMC6709911; doi:10.1371/journal.pone.0221500)
Supplement: S1 Table — (PDF) [file pone.0221500.s006.pdf]

S1 Table. Definition of inclusion and exclusion criteria

	Data source	Note
**Inclusion criteria**		
Warfarin	NorPD	ATC code B01AA03
Dabigatran	NorPD	ATC code B01AE07
Rivaroxaban	NorPD	ATC code B01AF01
Apixaban	NorPD	ATC code B01AF02
Atrial fibrillation	NorPD	Reimbursement code I48 (ICD-10) or K78 (ICPC-2) on first oral anticoagulant dispensing
**Exclusion criteria**		
Mitral stenosis	NPR	ICD-10 diagnosis code I05.x
Heart valve prosthesis	NPR	ICD-10 diagnosis code Z95.2, Z95.3 or Z95.4
Differing OACs dispensed on index date	NorPD	
Differing reimbursement codes if same OAC dispensed more than once on index date	NorPD	
Age <18 on index date	NR	
Uncertain resident status	NR	

ICD-10: International Classification of Diseases, 10th revision
ICPC-2: International Classification of Primary Care, 2nd Edition
NPR: Norwegian Patient Registry
NorPD: Norwegian Prescription Database
NR: National Registry
