# Supplementary material for: Comparative effectiveness of warfarin, dabigatran, rivaroxaban and apixaban in non-valvular atrial fibrillation: A nationwide pharmacoepidemiological study
Source: PLoS One. 2019 Aug 26;14(8):e0221500. doi: 10.1371/journal.pone.0221500 (PMC6709911; doi:10.1371/journal.pone.0221500)
Supplement: S2 Table — (PDF) [file pone.0221500.s007.pdf]

S2 Table. Definition of study outcomes

Outcome	ICD-10 code in NPR/NCoDR	Cause of death	Hospitalization		Out-patient visit	
			Primary diagnosis	Secondary diagnosis	Primary diagnosis	Secondary diagnosis
**Ischemic stroke, transient ischemic attack or systemic embolism**	Composite of ischemic stroke, transient ischemic attack and systemic embolism as defined below					
**Ischemic stroke or systemic embolism**	Composite of ischemic stroke and systemic embolism as defined below					
**Ischemic stroke**	I63.x I64.x	x	x	x		
**Transient ischemic attack**	G45.x		x	x		
**Systemic embolism**	I74.x	x	x	x		
**Major or clinically relevant non-major bleeding**	Combined outcome of intracranial, gastrointestinal and other bleeding as defined below					
**Intracranial bleeding**	I60.x I61.x I62.x S06.4 S06.5 S06.6	x	x	x		
**Gastrointestinal bleeding**	I85.0 I98.3 K25.0 K25.2 K25.4 K25.6 K26.0 K26.2 K26.4 K26.6 K27.0 K27.2 K27.4 K27.6 K28.0 K28.2 K28.4 K28.6 K29.0 K62.5 K92.0 K92.1 K92.2	x	x	x	x	x
**Other bleeding**	D62.x D68.3 H31.3 H35.6 H43.1 H45.0 I23.0 I31.2 J94.2 K66.1 M25.0 N42.1 N83.6 N83.7 N85.7 N89.7 N92.x N93.x N95.0 R04.x R31.x R58.x	x	x	x	x	x
**Pneumonia**	A48.1 J10.0 J11.0 J12.x J13.x J14.x J15.x J16.x J17.x J18.x J85.1	x	x	x		

Codes from the Norwegian Patient Register (NPR) includes both primary and secondary diagnoses from hospital admissions (and/or outpatient visits if specified). Codes from the Norwegian Cause of Death Register (NCoDR) include those registered as the underlying cause of death.
