# Supplementary material for: Comparative effectiveness of warfarin, dabigatran, rivaroxaban and apixaban in non-valvular atrial fibrillation: A nationwide pharmacoepidemiological study
Source: PLoS One. 2019 Aug 26;14(8):e0221500. doi: 10.1371/journal.pone.0221500 (PMC6709911; doi:10.1371/journal.pone.0221500)
Supplement: S3 Table — (PDF) [file pone.0221500.s008.pdf]

S3 Table. Definition of covariates

Variable	Norwegian Patient Registry	Norwegian Prescription Database		
	ICD-10 codes	Reimbursement codes		ATC codes
		ICD-10 codes	ICPC-2 codes	
**Medical history (last 5 years)**				
§Congestive heart failure	I11.0 I42.x I50.x J81.x	I11.0 I42 I50 I50.1 J81	K77	
§Hypertension	I10 I11.x I12.x I13.x I15.x O10.x O11.x	I10 I11 I11.0 I12 I13 I15 O10 O11	K86 K87	
§Diabetes mellitus	E10.x E11.x E13.x E14.x O24.0 O24.1 O24.3	E10 E10.2 E11 E112 E13 E14	T89 T90	
§Previous ischemic stroke, TIA or systemic embolism	G45.x I63.x I64.x I69.3 I69.4 I74.x	G45 I63 I74	K89 K90	
§History of acute myocardial infarction	I21.x I22.x I23.x	-22 I21 I22	-22 K75	
§Atherosclerosis or peripheral artery disease	I70.x I73.9	I70 I70.2 I73.9	K92	
§History of intracranial bleeding	I60.x I61.x I62.x I69.0 I69.1 I69.2 S06.4 S06.5 S06.6			
§History of gastrointestinal bleeding	I85.0 I98.3 K22.6 K25.0 K25.2 K25.4 K25.6 K26.0 K26.2 K26.4 K26.6 K27.0 K27.2 K27.4 K27.6 K28.0 K28.2 K28.4 K28.6 K29.0 K62.5 K92.0 K92.1 K92.2			
§History of other bleeding	D62 D68.3 H31.3 H35.6 H43.1 H45.0 I23.0 I31.2 J94.2 K66.1 M25.0 N42.1 N83.6 N83.7 N85.7 N89.7 N92.0 N92.1 N92.3 N92.4 N93.x N95.0 R04.x R31 R58			
§Liver disease	B17.0 B18.x I85.x I86.4 I98.2 I98.3 K70.x K71.x K72.x K73.x K74.x K75.x K76.x K77.x T86.4 Z94.4	K70 K71 K72 K74 K754 K76	D97	
§Chronic kidney disease	I12.x I13.x N00.x N01.x N02.x N03.x N04.x N05.x N07.x N11.x N14.x N17.x N18.x N19 N25.0 Q61.x T86.1 Z49.x Z94.0 Z99.2	I12 I13 N00 N01 N03 N04 N05 N11 N14 N18 N25 Q61 Z99.2	U88	
§Alcohol misuse	E24.4 F10.x G31.2 G62.1 G72.1 I42.6 K29.2 K70.x K85.2 K86.0 O35.4 P04.3 R78.0 T51.x Y90.x Y91.x Z50.2 Z71.4 Z72.1	F10.5 K70	P15	N07BB (÷N07BB04)
Other valvular heart disease	I06.x I07.x I08.x I34.x I35.x I36.x I37.x I38.x I39.x	I34 I35 I36 I37	K83	
Pacemaker, defibrillator	Z45.0 Z95.0			
Disease in precerebral or cerebral artery	I65.x I66.x I67.x I72.0 I72.5 I72.6 Q28.0 Q28.1 Q28.2 Q28.3	I65 I66 I67	K91	
Venous thromboembolism	I26.x I80.1 I80.2 I80.3 I80.8 I80.9 I81 I82.x	I26 I80 I81 I82	K93 K94	
Coagulation / platelet defects	D65 D66 D67 D68.x D69.x	D65 D66 D67 D68 D68.1 D68.2 D68.3	B83	

		D68.4 D68.8 D68.9 D69 D69.1 D69.3		
Esophagitis, gastritis, duodenitis, acid reflux or peptic ulcer	K20 K21.x K22.1 K22.7 K25.x K26.x K27.x K28.x K29.x	K20 K21 K25 K26	D84 D85 D86	
Inflammatory bowel disease	K50.x K51.x	K50 K51 K51.2	D94	
Anemia	D5* D60.x D61.x D62 D63.x D64.x	D51 D59	B81 B82	
Undernourished or vitamin deficiency	E4* E5* E60 E61 E63 E64	E53.8	T91	
Osteoporosis	M80.x M81.x M82.x	M80 M81	L95	
Fall	W0n.x			
Delirium	F05.x			
Dementia	F00.x F01.x F02.x F03.x F05.1 G30.x G31.1			N06DA02 N06DA03 N06DA04 N06DX01
Cancer	C* (÷C44.x) D45			
Chronic pulmonary disease	I27.x J4* J70.x J84.x J92.x J96.x E66.2 J98.2 J98.3	I27 J40 J41 J42 J43 J44 J45 J47 J84 J84.1	K82 R79 R95 R96	
**Prescription drug use (last 6 months)**				
§Nonsteroidal anti-inflammatory drug				M01A N02BA01
§Platelet aggregation inhibitor or acetylsalicylic acid				B01AC
Angiotensin converting enzyme (ACE) inhibitor or angiotensin II antagonist				C09A C09B C09C C09D
Antiarrhythmic				C01B
Beta blocking agent				C07
Calcium channel blocker				C08C C08D
Digitoxin / digoxin				C01AA04 C01AA05
Diuretics				C03A C03C C03D C03E
Lipid modifying agent				C10
Nitrate				C01DA
Antacids, H2-receptor antagonist or proton pump inhibitor				A02A A02BA A02BC
Glucocorticoids				H02AB
Antidepressant				N06A
Antipsychotic				N05A
Anxiolytic, hypnotic or sedative				N05B N05C
Antiepileptic				N03A

**Other characteristics**	**Source**	**Note**
§Age	National Registry	Only month and year of birth available – assumed that all participants were born on day 15 of birth month.
County of residence	National Registry	Østfold, Akershus, Oslo, Hedmark, Oppland, Buskerud, Vestfold, Telemark, Aust-Agder, Vest-Agder, Rogaland,

		Hordaland, Sogn og Fjordane, Møre og Romsdal, Sør-Trøndelag, Nord-Trøndelag, Nordland, Troms, Finnmark
§Sex	National Registry	
OAC initiated at hospital	Norwegian Prescription Database	ICD10 reimbursement code for atrial fibrillation (I48) on first oral anticoagulant dispensing.<br>(Primary care physicians use ICPC2 reimbursement codes.)
§Year OAC initiated	Norwegian Prescription Database	
Number of drugs dispensed last 6 months	Norwegian Prescription Database	0-4, 5-9, 10-14, 15-
Number of hospital admissions last 6 months	Norwegian Patient Registry	0, 1, 2-
Number of outpatient visits last 6 months	Norwegian Patient Registry	0, 1, 2-

§: Covariates used in partially adjusted model
ICD-10: International Classification of Diseases, 10th revision
ICPC-2: International Classification of Primary Care, 2nd Edition
TIA: Transient ischemic attack
OAC: Oral anticoagulation
