# Supplementary material for: Comparative effectiveness of warfarin, dabigatran, rivaroxaban and apixaban in non-valvular atrial fibrillation: A nationwide pharmacoepidemiological study
Source: PLoS One. 2019 Aug 26;14(8):e0221500. doi: 10.1371/journal.pone.0221500 (PMC6709911; doi:10.1371/journal.pone.0221500)
Supplement: S4 Table — (PDF) [file pone.0221500.s009.pdf]

S4 Table. Calculation of CHA_2DS_2-VASc and modified HAS-BLED risk scores from variables in S3 Table

Risk score	Variable	Points
**CHA_2DS_2-VASc**	Congestive heart failure	1
	Hypertension	1
	Age ≥75 years	2
	Diabetes mellitus	1
	Previous ischemic stroke, TIA or systemic embolism	2
	History of acute myocardioal infarction or atherosclerosis/peripheral vascular disease	1
	Age 65-74 years	1
	Female sex	1
**Modified HAS-BLED**	Hypertension	1
	Abnormal liver function	1
	Abnormal renal function	1
	Previous ischemic stroke, TIA or systemic embolism	1
	History of intracranial, gastrointestinal or other bleeding	1
	Age >65 years	1
	Concomitant use of NSAID, ASA or platelet aggregation inhibitor	1
	Alcohol misuse	1
