# Supplementary material for: Comparative effectiveness of warfarin, dabigatran, rivaroxaban and apixaban in non-valvular atrial fibrillation: A nationwide pharmacoepidemiological study
Source: PLoS One. 2019 Aug 26;14(8):e0221500. doi: 10.1371/journal.pone.0221500 (PMC6709911; doi:10.1371/journal.pone.0221500)
Supplement: S5 Table — (PDF) [file pone.0221500.s010.pdf]

S5 Table. Results of sensitivity analyses on primary effectiveness outcome

Outcome	Model	Oral anticoagulant	Median days of follow-up (min-max)	No. of events	Incidence rate (per 100 person years)	Hazard ratio (95% confidence interval)		
						Crude	Partially adjusted	Fully adjusted
Ischemic stroke, transient ischemic attack or systemic embolism	rWTD 50th percentile interarrival density[a]	Warfarin	114 (1-365)	85	3.34	1	1	1
		Dabigatran	97 (1-365)	68	3.63	0.95	1.03 (0.74-1.43)	1.05 (0.74-1.49)
		Rivaroxaban	89 (1-365)	99	4.73	1.18	1.13 (0.84-1.52)	1.14 (0.84-1.56)
		Apixaban	54 (1-365)	108	6.23	1.21	1.12 (0.82-1.52)	1.04 (0.75-1.44)
	rWTD 60th percentile interarrival density[a]	Warfarin	138 (1-365)	93	3.15	1	1	1
		Dabigatran	118 (1-365)	74	3.21	0.93	1.01 (0.74-1.38)	1.04 (0.74-1.44)
		Rivaroxaban	96 (1-365)	109	4.24	1.17	1.13 (0.85-1.50)	1.16 (0.86-1.56)
		Apixaban	63 (1-365)	114	5.39	1.13	1.03 (0.77-1.39)	0.98 (0.71-1.34)
	rWTD 70th percentile interarrival density[a]	Warfarin	169 (1-365)	102	3.02	1	1	1
		Dabigatran	143 (1-365)	78	2.81	0.88	0.97 (0.72-1.31)	0.99 (0.72-1.35)
		Rivaroxaban	112 (1-365)	119	3.71	1.12	1.11 (0.84-1.45)	1.11 (0.84-1.48)
		Apixaban	76 (1-365)	133	4.90	1.15	1.07 (0.81-1.41)	1.01 (0.75-1.35)
	rWTD 80th percentile interarrival density[a]	Warfarin	204 (1-365)	110	2.91	1	1	1
		Dabigatran	171 (1-365)	85	2.67	0.88	0.99 (0.74-1.32)	1.02 (0.75-1.38)
		Rivaroxaban	138 (1-365)	131	3.57	1.14	1.14 (0.88-1.47)	1.15 (0.88-1.51)
		Apixaban	102 (1-365)	158	4.14	1.15	1.09 (0.84-1.41)	1.03 (0.78-1.36)
	rWTD 99th percentile interarrival density[a]	Warfarin	322 (1-365)	137	3.00	1	1	1
		Dabigatran	288 (1-365)	95	2.38	0.77	0.88 (0.67-1.15)	0.93 (0.70-1.22)
		Rivaroxaban	205 (1-365)	157	3.48	1.08	1.08 (0.86-1.37)	1.13 (0.88-1.44)
		Apixaban	203 (1-365)	206	3.47	1.06	1.01 (0.81-1.28)	1.00 (0.78-1.27)
	Fixed daily dose model[b]	Warfarin	167 (1-365)	100	2.93	1	1	1
		Dabigatran	215 (1-365)	86	2.40	0.86	0.97 (0.72-1.30)	1.02 (0.75-1.39)
		Rivaroxaban	223 (1-365)	173	3.66	1.32	1.27 (0.99-1.63)	1.33 (1.02-1.72)
		Apixaban	174 (1-365)	193	3.53	1.20	1.11 (0.86-1.44)	1.09 (0.83-1.42)
	Only main diagnosis events[c]	Warfarin	250 (1-365)	98	2.35	1	1	1
		Dabigatran	212 (1-365)	61	1.71	0.70	0.83 (0.60-1.15)	0.81 (0.57-1.14)
		Rivaroxaban	165 (1-365)	95	2.35	0.93	0.95 (0.71-1.26)	0.92 (0.68-1.24)
		Apixaban	151 (1-365)	125	2.46	0.93	0.89 (0.67-1.18)	0.80 (0.60-1.08)

[a] Prescription length estimated in reverse parametric waiting time distribution (rWTD) model with 50th, 60th, 70th, 80th or 99th percentile interarrival density adjusted for sex, age and number of pills. No gap in supply allowed.

[b] Prescription length based on recommended dose for DOACs and an estimate of mean daily dose of warfarin used by the study population. Up to 30-day gap in supply allowed.

[c] Only events registered as primary diagnosis included in outcome (excluding events registered as secondary diagnosis or underlying cause of death). Otherwise same model as main analyses.
