# Supplementary material for: Comparative effectiveness of warfarin, dabigatran, rivaroxaban and apixaban in non-valvular atrial fibrillation: A nationwide pharmacoepidemiological study
Source: PLoS One. 2019 Aug 26;14(8):e0221500. doi: 10.1371/journal.pone.0221500 (PMC6709911; doi:10.1371/journal.pone.0221500)
Supplement: S6 Table — (PDF) [file pone.0221500.s011.pdf]

S6 Table. Results of sensitivity analyses on primary safety outcome

Outcome	Model	Oral anticoagulant	Median days of follow-up (min-max)	No. of events	Incidence rate (per 100 person years)	Hazard ratio (95% confidence interval)		
						Crude	Partially adjusted	Fully adjusted
Major or clinically relevant non-major bleeding	rWTD 50th percentile interarrival density[a]	Warfarin	111 (1-365)	331	13.28	1	1	1
		Dabigatran	96 (1-365)	137	7.36	0.51	0.67 (0.54-0.82)	0.69 (0.56-0.86)
		Rivaroxaban	89 (1-365)	246	11.88	0.81	0.94 (0.79-1.12)	0.96 (0.80-1.15)
		Apixaban	54 (1-365)	179	10.36	0.65	0.71 (0.58-0.86)	0.67 (0.54-0.83)
	rWTD 60th percentile interarrival density[a]	Warfarin	134 (1-365)	379	13.14	1	1	1
		Dabigatran	117 (1-365)	168	7.33	0.53	0.69 (0.57-0.83)	0.70 (0.58-0.86)
		Rivaroxaban	96 (1-365)	287	11.32	0.81	0.94 (0.80-1.10)	0.97 (0.82-1.14)
		Apixaban	63 (1-365)	215	10.21	0.66	0.72 (0.60-0.86)	0.69 (0.57-0.83)
	rWTD 70th percentile interarrival density[a]	Warfarin	163 (1-365)	422	12.84	1	1	1
		Dabigatran	141 (1-365)	197	7.16	0.54	0.69 (0.58-0.82)	0.70 (0.58-0.84)
		Rivaroxaban	109 (1-365)	331	10.50	0.79	0.91 (0.79-1.06)	0.93 (0.80-1.09)
		Apixaban	76 (1-365)	254	9.42	0.64	0.69 (0.58-0.81)	0.66 (0.55-0.78)
	rWTD 80th percentile interarrival density[a]	Warfarin	196 (1-365)	464	12.67	1	1	1
		Dabigatran	168 (1-365)	225	7.15	0.56	0.70 (0.60-0.82)	0.72 (0.61-0.85)
		Rivaroxaban	131 (1-365)	381	10.59	0.82	0.94 (0.82-1.08)	0.96 (0.83-1.11)
		Apixaban	101 (1-365)	347	9.18	0.67	0.71 (0.61-0.83)	0.69 (0.59-0.81)
	rWTD 99th percentile interarrival density[a]	Warfarin	301 (1-365)	531	12.05	1	1	1
		Dabigatran	276 (1-365)	270	6.88	0.57	0.71 (0.61-0.82)	0.72 (0.62-0.85)
		Rivaroxaban	194 (1-365)	465	10.54	0.85	0.96 (0.84-1.09)	0.96 (0.84-1.10)
		Apixaban	197 (1-365)	501	8.57	0.69	0.70 (0.62-0.80)	0.68 (0.59-0.78)
	Fixed daily dose model[b]	Warfarin	160 (1-365)	412	12.42	1	1	1
		Dabigatran	209 (1-365)	247	6.99	0.58	0.70 (0.60-0.82)	0.73 (0.62-0.86)
		Rivaroxaban	214 (1-365)	515	11.14	0.92	0.98 (0.86-1.12)	1.01 (0.88-1.16)
		Apixaban	169 (1-365)	466	8.66	0.70	0.71 (0.61-0.81)	0.69 (0.59-0.80)
	Only main diagnosis events[c]	Warfarin	242 (1-365)	333	8.17	1	1	1
		Dabigatran	207 (1-365)	178	5.05	0.61	0.71 (0.59-0.86)	0.72 (0.60-0.88)
		Rivaroxaban	158 (1-365)	294	7.40	0.88	0.96 (0.82-1.13)	0.97 (0.81-1.14)
		Apixaban	149 (1-365)	300	5.97	0.70	0.72 (0.61-0.85)	0.69 (0.58-0.83)

[a] Prescription length estimated in reverse parametric waiting time distribution (rWTD) model with 50th, 60th, 70th, 80th or 99th percentile interarrival density adjusted for sex, age and number of pills. No gap in supply allowed.

[b] Prescription length based on recommended dose for DOACs and an estimate of mean daily dose of warfarin used by the study population. Up to 30-day gap in supply allowed.

[c] Only events registered as primary diagnosis included in outcome (excluding events registered as secondary diagnosis or underlying cause of death). Otherwise same model as main analyses.
