# Supplementary material for: Comparative effectiveness of warfarin, dabigatran, rivaroxaban and apixaban in non-valvular atrial fibrillation: A nationwide pharmacoepidemiological study
Source: PLoS One. 2019 Aug 26;14(8):e0221500. doi: 10.1371/journal.pone.0221500 (PMC6709911; doi:10.1371/journal.pone.0221500)
Supplement: S7 Table — (PDF) [file pone.0221500.s012.pdf]

S7 Table. Results of sensitivity analysis on negative control outcome

Outcome	Model	Oral anticoagulant	Median days of follow-up (min-max)	No. of events	Incidence rate (per 100 person years)	Hazard ratio (95% confidence interval)		
						Crude	Partially adjusted	Fully adjusted
**Pneumonia**	**Negative control[a]**	Warfarin	243 (1-365)	306	7.48	1	1	1
		Dabigatran	210 (1-365)	148	4.18	0.55	0.79 (0.65-0.97)	0.91 (0.74-1.12)
		Rivaroxaban	162 (1-365)	194	4.83	0.62	0.80 (0.66-0.96)	0.85 (0.70-1.03)
		Apixaban	149 (1-365)	324	6.45	0.80	0.90 (0.77-1.07)	0.91 (0.76-1.09)

[a] Same model as the main analyses with hospitalization or death from pneumonia as a negative control outcome to indirectly test for residual confounding.
